# Structural Insights into the Design of Synthetic Nanobody Libraries

**DOI:** 10.3390/molecules27072198

**Published:** 2022-03-28

**Authors:** Mario S. Valdés-Tresanco, Andrea Molina-Zapata, Alaín González Pose, Ernesto Moreno

**Affiliations:** 1 Faculty of Basic Sciences, University of Medellin, Medellin 050026, Colombia; amz-129@outlook.com (A.M.-Z.); alaingonzalez29@gmail.com (A.G.P.); 2 Grupo de Micología Médica y Experimental, Corporación para Investigaciones Biológicas (CIB), Medellin 050034, Colombia

**Keywords:** nanobody, synthetic library, phage display, ribosome display, rational design

## Abstract

Single domain antibodies from camelids, or nanobodies, are a unique class of antibody fragments with several advantageous characteristics: small monomeric size, high stability and solubility and easy tailoring for multiple applications. Nanobodies are gaining increasing acceptance as diagnostic tools and promising therapeutic agents in cancer and other diseases. While most nanobodies are obtained from immunized animals of the camelid family, a few synthetic nanobody libraries constructed in recent years have shown the capability of generating high quality nanobodies in terms of affinity and stability. Since this synthetic approach has important advantages over the use of animals, the recent advances are indeed encouraging. Here we review over a dozen synthetic nanobody libraries reported so far and discuss the different approaches followed in their construction and validation, with an emphasis on framework and hypervariable loop design as critical issues defining their potential as high-class nanobody sources.

## 1. Introduction

**Figure 1:**
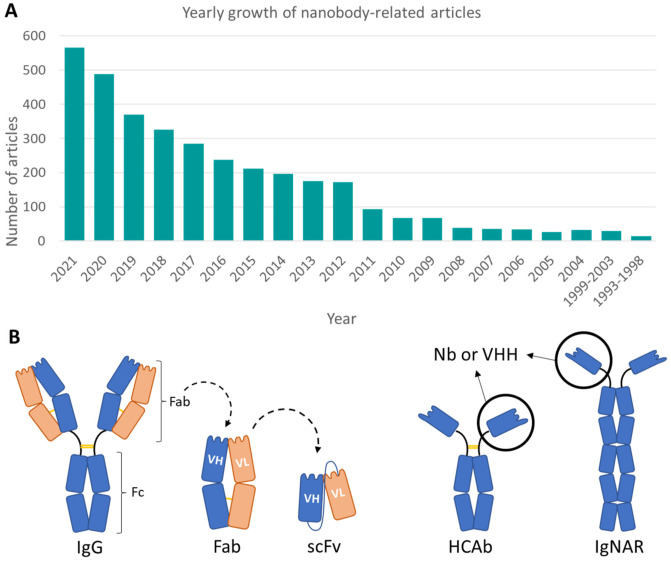
(**A**) Articles per-year based on a “nanobody” search in PubMed. (**B**) Differences between traditional antibodies and their derivatives with respect to HCAbs. A classical IgG antibody is made up of 12 immunoglobulin domains, distributed in two different pairs of chains: heavy (in blue) and light (in orange). It is functionally divided into the Fc region and the two Fab fragments, where the recognition region is located. On the other hand, both HCAbs and IgNARs are made up of one pair of a single chain type. Different antibody fragments, such as the Fab or a single-chain fragment (scFv) can be derived or constructed. The latter is formed by the heavy and light variable domains, joined by a linker. In contrast, Nbs or VHH are formed solely by the recognition domain of HCAbs or IgNARs.

Since the discovery in 1993 of heavy-chain antibodies (HCAbs) [1] and the antibody fragments derived from them, called single domain antibodies (sdAbs) or nanobodies (Nbs), the number of studies related to these antibody fragments has been exponentially increasing every year (Figure 1A). Recently, the Food and Drug Administration (FDA) approved the first Nb for therapeutic use: caplacizumab, for the treatment of acquired thrombotic thrombocytopenic purpura [2]. More recently, during the current pandemic, a considerable number of possible solutions based on Nbs have been generated against SARS-CoV-2 [3,4].

HCAbs, from which Nbs are derived, are found in members of the Camelidae family that includes camels, dromedaries, alpacas, and llamas. Structurally, Nbs are made up of a single immunoglobulin domain with a molecular weight of ~15 KDa, being smaller and more compact than the smallest classical antibody fragment—the single-chain variable fragment (scFv) [5] (Figure 1B).

Nbs have a unique set of advantages over antibodies. Their small size, hydrophilic nature, stability and resistance to reducing environments, allow their production in different expression systems, such as bacteria, yeast, or mammalian cells. Remarkably, despite their smaller binding region, Nbs can achieve affinities in the nanomolar order, similar to those reported for antibodies. Furthermore, their modularity allows the generation of multivalent constructs, fusion with other molecules, functionalization of nanoparticles and many other constructs [6].

Antigen-specific Nbs are obtained mainly from three types of genetic sources: immune, naïve and synthetic libraries. Both immune and naïve library generation requires animal components, with immune libraries being the main Nb source. Synthetic libraries, on the other hand, are emerging as an attractive alternative to circumvent animal use [7]. Here we review the development of synthetic nanobody libraries, discussing the different approaches followed in their construction and validation, with an emphasis on the framework and hypervariable loop design as critical issues defining their potential as high-class nanobody sources.

## 2. Structural Bases for the Design of Synthetic Nb Libraries

Synthetic libraries comprise synthetic and semi-synthetic libraries. They differ mainly in the level of design involved in their construction. Structurally, nanobodies can be divided into two relevant parts: the framework and the hypervariable loops, also known as complementarity determining regions (CDRs). In constructing a semisynthetic library, a previously characterized Nb is used as starting point and then individual CDRs, usually only CDR3, are randomized using an ad-hoc design [8,9,10,11]. Building a synthetic library, though, requires a more elaborate design, in which the framework region requires special attention, as we discuss below in this section. While a wide variety of immune Nb libraries are found in the literature, the number of synthetic and semisynthetic libraries has been growing only in recent years (Table 1).

### 2.1. Key Features for Framework Selection

An obligatory reference when reviewing the development of synthetic Nb libraries is the pioneering work by Saerens and co-workers [24], describing the design and validation of one of the first “universal” frameworks: cAbBCII10, derived from an Nb against Beta-Lactamase II, previously reported by Conrath and co-workers [25]. This framework was selected for its high stability, good levels of expression in bacteria and its remarkable capability of remaining functional even in the absence of the canonical disulfide bridge [24]. Furthermore, this framework is highly plastic, which they validated from the grafting of CDRs 1 and 3 of different lengths from various functional Nbs. In all cases, the obtained Nbs showed recognition of their respective antigens, as well as an increase in their thermostability [24]. Notably, the cAbBCII10 framework continues to be widely used in the design of new synthetic libraries.

**Figure 2:**
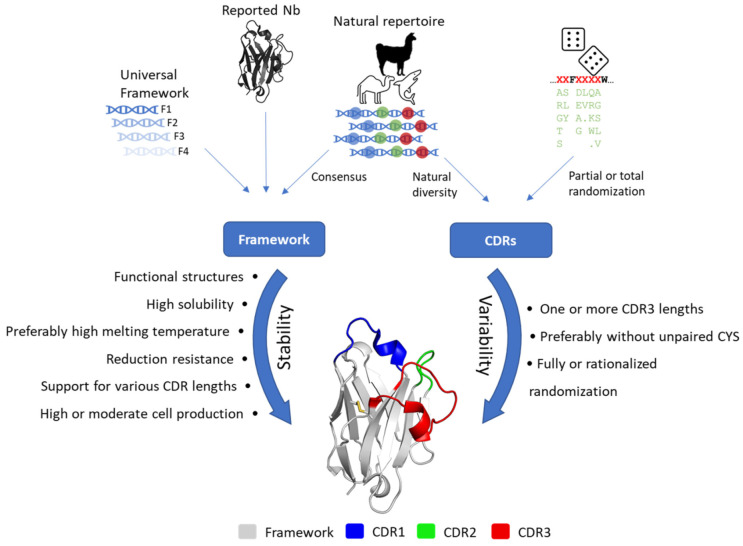
Strategy and desired physicochemical characteristics for the generation of synthetic libraries. The design and construction of a synthetic library comprise two main factors: the framework and the CDRs. A previously reported universal or Nb-derived framework can be used for a new library. Alternatively, a new framework can be generated from a consensus sequence derived from natural repertoires. Similarly, the design of CDRs can be carried out in a rationalized way, considering the natural diversity in natural repertoires, or be generated in a partially or completely randomized way. While framework selection is more focused on stability, CDR design focuses more on variability. In both cases, the listed characteristics should be considered to obtain a robust and functional synthetic library.

Selecting a nanobody framework capable of supporting a large diversity of hypervariable loops is a challenging task. The strategies for obtaining a versatile and robust framework are varied, as illustrated below in the section describing a few selected synthetic nanobody libraries. In this section, we discuss important points to be considered when selecting or designing a universal Nb framework. Such a framework must meet several key properties [24,26,27,28,29,30], represented in Figure 2.

Different strategies can be applied in designing or selecting a suitable framework for a synthetic library. In recent works, framework design was based on consensus sequences derived from different sets of nanobodies [12,15,17]. However, the construction resulting from such a consensus might not be endowed with the desired properties and, therefore, would need a comprehensive experimental characterization. Thus, the most common, simpler approach has been to use a previously characterized framework [24]. Typically, framework functionality is evaluated from a grafting experiment consisting in transplanting the CDRs (or just CDR3) from a functional Nb, specific for a particular antigen, onto the framework of interest. Then, the ability of the new construct to recognize the same antigen, as well as other functional properties are evaluated [12,24,29].

Solubility is another important factor to be considered since it may affect the production and long-term stability of the selected nanobodies. Poor solubility results in the formation of high molecular weight molecular aggregates, which causes precipitation [30,31]. Furthermore, it affects protein functionality by preventing the diffusion and binding of Nbs to their antigens [31,32,33].

The melting temperature (Tm), defined as the temperature at which 50% of the molecules become denatured, is another fundamental factor in processes of molecular modification, storage, and industrial production, not only for Nbs but for proteins in general [33]. Chemical conjugation of Nbs with chelated metals and fluorophores are reactions commonly used in diagnostic solutions, or in the functionalization of nanoparticles for controlled drug release, just to name a few examples. For several of these chemical reactions, a moderate or high thermal resistance is required for the Nb, to avoid denaturation. In this regard, favorable denaturation-refolding dynamics become important to compensate for possible denaturation processes. Unfolding reversibility is evaluated by estimating both the refolding ratio and the retention of Nb functionality after a gradual temperature decrease. This property becomes more important the lower the Tm, especially for Nbs derived from immune or naive libraries, for which the chance of improving Tm without affecting affinity and/or specificity is small [28,30,34,35].

Several studies have shown that introducing non-canonical disulfide bridges contributes considerably to the stability of Nbs and increases the Tm [36,37,38,39], although extra disulfide bridges may considerably affect the expression levels of the selected Nbs. Nonetheless, several strategies can be developed to overcome this problem [30]. By combining such protein engineering with selection under protease pressure, it is possible to obtain Nbs that are in addition resistant to proteolytic degradation [35].

On the other hand, stability in a reducing environment is essential to withstand several chemical modifications that require reducing conditions. Beyond diagnostic and therapeutic applications, this characteristic is essential for the design and production of the so-called intrabodies, which recognize intracellular molecules, and therefore, should be capable of functioning in the cytoplasm [40,41,42]. This research field is gaining special relevance because of the great potential of intrabodies, for example, to characterize and modulate intracellular physiological processes and to inhibit the function of cytoplasmic targets [40,42,43,44,45]. Likewise, stability in a reducing environment is essential for expression in the cellular cytoplasm of bacteria and eukaryotic cells [46].

Several reported Nb libraries were designed with each CDR having a fixed length [16,18,19,20,21,47]. In more recent works, however, libraries comprising various CDR3 lengths have been constructed in order to increase their structural variability [12,13,14,15,17]. Therefore, the selected framework must be capable of supporting different CDR3 structures, remaining functional and stable. Various studies have found a correlation between the length and composition of CDRs, especially CDR3, with characteristics, such as Tm, solubility and expression levels, although only a moderate correlation has been reported in several cases, which points to the framework structure as the most relevant factor [48,49].

Another important feature to consider is the level of cellular production of the recombinant Nbs obtained from the library. Although there are currently various protocols and expression systems focused on improving the production of recombinant proteins [50,51,52], starting from a framework with proven high cellular expression may be a safer approach. At the laboratory scale, expression levels must be sufficient to allow proper purification and characterization. If the Nb needs to be produced for practical applications that demand larger quantities, then this factor becomes even more relevant. One of the strategies that have been applied to improve the cellular expression of Nbs is performing point mutations at certain positions of the framework [26,30].

Last but not least, low immunogenicity is required for nanobodies that are intended for therapeutic applications or recurrent in vivo imaging in humans. This requirement, however, is not necessary if the Nb is directed to external diagnosis or is intended for research or industrial applications. Aiming to lower potential immunogenicity, the Nb framework may be subjected to a humanization procedure, consisting in substituting certain residues by amino acids commonly found in the corresponding positions of the heavy chains of human antibodies [53]. This process is challenging, as humanization might affect the stability of the framework. On the other hand, since immunogenicity is measured in preclinical and clinical stages, it is difficult to know in advance the actual weight this factor might have for the final therapeutic product. At this point, it is worth noting that several chimeric antibodies, that is, antibodies that retain the original mouse variable regions fused with constant human regions, have been successfully used in the clinics for many years (e.g., Cetuximab [54,55]) despite their partial xenogeneic composition.

### 2.2. Design of the Hypervariable Loops

CDR design is based on different assumptions regarding the variability to be introduced in each of the three loops. Although in most cases all CDRs interact with the antigen [56,57], CDR3 is undoubtedly the most important, being the loop that most frequently interacts with the antigen and also the most variable, both in length and amino acid composition. The lengths of CDRs 1 and 2 remain between 5 and 8 amino acids, whereas CDR3 length varies between 6 and 18 amino acids [57,58].

The most common strategy in the design of the hypervariable regions is to leave CDR1 and CDR2 at a fixed length each, whereas CDR3 may be represented by several different lengths, especially in the most recently reported libraries [12,13,14,15,17,18,19,20,22,23]. In most cases, the variability of CDRs 1 and 2 is set to represent the amino acid variability found in natural Nb repertoires. For CDR3, in contrast, the common approach is to completely randomize each position, allowing the occurrence of all amino acids except cysteine, which is usually excluded to avoid the formation of dimers, aggregation, or folding problems [12,13,15]. In two recent studies, however, CDR3 was rationally designed, preserving a set of amino acids with certain properties [13,14].

Together with the framework, the hypervariable region has an important influence on the physicochemical properties of the Nbs. Nonetheless, CDR design always tries to maximize variability. Several studies have related the length and composition of CDR3 to thermostability, solubility, expression levels in bacterial cells and, to a lesser extent, the type of antigen the Nb is directed to [14,48,49,59]. The latter factor was taken into account by Zimmermann et al. [14], who created three different libraries based on the observed shapes of hypervariable regions, using three different CDR3 lengths: 6 aa (concave shape), 12 aa (loops) and 16 aa (convex shape).

## 3. Advantages and Limitations of Synthetic Libraries

### 3.1. Advantages

Even though the use of synthetic libraries is still challenging, the benefits it brings have fueled its steady growth. As described by Muyldermans in an excellent review [7], there are many advantages to using synthetic libraries (Table 1):

*-Do not require immunization or animal components*. This not only means avoiding experimentation with animals, a topic of special relevance today but provides also other advantages. Firstly, raising experimental animals under special conditions carries a high cost. Secondly, the immunization process is long, tedious and involves several animals in order to obtain a broad Nb repertoire. Although naïve libraries do not either require immunization, the procedure is complex and involves animals. In particular, to obtain a library similar in size to a typical synthetic library, a few liters of blood collected from at least 10 animals is required [7,60,61].

-*Works for non-immunogenic or toxic targets.* Several molecules, such as RNA or DNA are not immunogenic or at least fail to elicit an immune response in the HCAb classes, while other compounds might be too toxic, too contagious, or too harmful for animals. In such cases, suitable nanobodies may be selected from a naïve or a synthetic Nb repertoire [7].

-*Can be used in multiple projects*. A library obtained by animal immunization is enriched in antigen-specific Nbs due to the affinity maturation process. Synthetic libraries, on the other hand, are nonspecific and seek to recreate the largest possible variability. That is why the number of Nb binders and their affinity values are generally higher in immune libraries [7]. However, recent works have shown that synthetic libraries can yield high affinity Nbs against different antigens, comparable to those obtained from immune libraries [12,14,20,22].

-*Shorter time to obtain Nb binders*. Once the synthetic Nb library has been designed and synthesized, it can be used with many different antigens at a time, furthermore, the binder selection process is greatly shortened. While the animal immunization process typically takes about two months, the selection of binders from synthetic libraries may take between two and three weeks [7].

-*Optimized physicochemical properties.* Using a framework with the characteristics described above would produce Nbs with desired physicochemical properties, high levels of cellular production and high thermostability, among other favorable properties [12,14]. For Nbs selected from conventional libraries based on specificity and affinity, these physicochemical properties might be absent and would be difficult to engineer.

### 3.2. Limitations

It is necessary to consider a few possible obstacles that may arise when using a synthetic Nb library:

-*The selected Nb might have weakened physicochemical properties*. The framework-CDR combination of the selected Nb might have a lower stability/solubility. This possibility, however, is minimized with an optimal framework selection, as discussed above.

-*Large libraries are required*. The size of the library is determined by the number of transformants that can be obtained. To increase the probability of obtaining good binders, synthetic libraries must be large (at least 10^8^ in size, preferably larger) [7]. In contrast, immune libraries have a size typically within a range of 10^6^–10^8^. Among other factors, it is the large variability and non-specificity which provide the library with flexibility for use in various projects.

-*The affinity may be lower than with immune libraries*. Nbs obtained from immune libraries are the product of affinity maturation, so the obtained repertoire is enriched toward the antigen of interest. Given the variability and non-specificity of synthetic libraries, the chances of obtaining high affinity Nbs are in principle lower. Nonetheless, recent studies have reported Nbs, selected from synthetic libraries, with affinities in the nanomolar range [12,14,20,22] and even in the subnanomolar order [12,14,20]. In addition, the affinity of a selected Nb may be further improved by an in vitro maturation process [15,62].

## 4. Selected Examples of Synthetic Nb Libraries

In this section, we recapitulate the design strategies applied in the construction of four synthetic and semi-synthetic libraries recently reported in the literature, as well as the different methods followed for their validation.

### 4.1. Moutel et al., (2016)

The development of the NaLi-H1 phage-displayed library [12] is probably one of the most comprehensive and detailed studies focused on the design and validation of a synthetic Nb library.

*Framework selection and validation*. Moutel and co-workers selected highly stable Nbs expressed in the bacterial cytoplasm from a set of clones previously selected from immune or llama naïve libraries. The selection was carried out from experiments in which the carboxy-terminus of the Nbs was fused to an HA-labeled chloramphenicol acetyltransferase (CAT). In these assays, only bacteria with functional fused Nbs can grow in an environment with a high antibiotic concentration. Based on the production levels in *E. coli*, as well as the apparent solubility of EGFP fusions in mammalian cells, the authors selected a consensus framework sequence, which matched the framework of the Nb with the best characteristics (sdAbD10). A humanized version of this framework (hs2dAb) was also constructed by substituting seven amino acids, out of 12 possible residues, by those most represented in the human VH3 family, while the so-called hallmark amino acids and Q103 were left unchanged, so as not to affect the stability of the original framework. Subsequently, the robustness of these two frameworks was validated by grafting experiments using the CDRs of an anti-lam1 VHH [63]. With both frameworks, the resulting Nbs were correctly presented in the phages, showed good production, and recognized the endogenous lam1 antigen while functioning as intrabodies.

*CDR design*. Both CDR1 and CDR2 were kept with a constant length of seven residues, using at each position a set of amino acids that partially recapitulates natural diversity. In addition, the likelihood of having hydrophobic amino acids was reduced to decrease the propensity for aggregation. For CDR3, all the positions were randomized, allowing all amino acids except cysteine. To cover the wide spectrum of natural variability found in camelid VHH domains, four different CDR3 lengths were used: 9, 12, 15 and 18 aa.

*Library construction*. The Nb genes were synthesized by trinucleotide DNA assembly and inserted into a modified pHEN2 plasmid, used to transform TG1 *E. coli* bacteria. As result, a library having a size of approximately 3 × 10^9^ individual recombinant clones was obtained, with an estimated fraction of potentially bad clones around 4%. Sequencing of a set of 5.6 × 10^6^ genes showed the expected diversity and statistical amino acid distribution in the three CDRs.

*Library validation*. First, the solubility and thermal stability were assessed for a group of 24 randomly selected clones. After being heated up to 90 °C, 70% of them recovered their solubility. To validate the diversity and quality of the library, Moutel and coworkers carried out a series of experiments aimed at the selection of Nbs against different antigens with varied characteristics and to prove their capabilities as intrabodies. Two of these antigens were the fluorescent proteins EGFP and mCherry, which allow easy detection. Tubulin and β-actin, which are constitutive proteins highly expressed in cells, were also included in the antigen set, as well as other two molecules: the tumor suppressor protein p53 (an 83 aa fragment) and HP1α, with low expression levels. In all cases, high affinity specific Nbs were recovered. Furthermore, for EGFP, mCherry, p53, HP1α and RHO GTPase the authors proved the antigen-specific binding of the obtained Nbs in the cytoplasm (Table 2).

Notably, the authors demonstrated a unique advantage of the in vitro selection with synthetic libraries by selecting a conformer-specific Nb against the active form of a GTP binding protein of the RHO family. Finally, they carried out a selection of Nbs against cell surface antigens. After subtractive selection, they obtained several clones capable of efficiently recognizing the extracellular region of the HER2 receptor on the cell surface (Table 2).

Remarkably, the affinity values of the Nbs obtained against this varied antigen panel ranged from 50 pM to 10 nM. These values, obtained for monovalent binders, are comparable to those obtained by immunization or in vitro affinity maturation.

### 4.2. McMahon et al., (2018)

McMahon and coworkers reported a new synthetic nanobody library that was designed following a structure-based approach [13]. A relevant point in this work is the development of a platform for displaying nanobodies or other proteins on the yeast surface, based on a newly designed amino acid tether to anchor the nanobodies to the yeast cell wall. Further interesting results are the selection of Nbs against a nonpurified antigen and Nbs specific for the active, antagonist-binding conformers of two protein receptors.

*Framework design*. A consensus framework derived from the llama genes IGHV1S1—IGHV1S1S5 was used for library construction.

*CDR design*. The design of the CDRs recapitulates the natural diversity of amino acids observed in the set of Nb crystal structures available at the time of the study (93 structures). In addition, a few framework positions adjacent to the CDRs were also partially randomized. Four and one positions in CDR1 and CDR2, respectively, showing the largest variability in the analyzed sequence set, were selected for full randomization. In all cases, also for CDR3, cysteine and methionine were avoided. Given the importance of CDR3 in antigen recognition, and its length and amino acid variation, this loop was constructed with three different lengths: 7, 11 and 15 aa., which were all fully randomized.

*Library construction*. The gene library was constructed using a two-step overlay and extension PCR (OE-PCR). The final estimated clonal size of the yeast-displayed library was approximately 1 × 10^8^. Analysis of 480,000 unique sequences confirmed that the frequency and variability of the randomized positions were as expected. For library creation, a designed 649–amino acid tether was used, including an N-terminal engineered mating factor α preprotein, which enhances antibody expression in yeast, together with a glycosylphosphatidylinositol anchor sequence at the C terminus, which covalently tethers the nanobody to the yeast cell wall. Most of the Nbs analyzed for library validation showed high production levels (>20 mg/L).

*Library validation*. The functional validation of this library was carried out by selecting Nbs against four antigens of different classes: two soluble—human serum albumin (HSA) and human adiponectin—and two membrane proteins—the β2 adrenergic receptor (β2AR) and the A2A adenosine receptor (A2AR) (Table 2). After four biopanning rounds, a highly specific Nb against HSA was obtained, although with a modest affinity (430 nM). The specificity was demonstrated by the capability of differentiating HSA from the closely related mouse serum albumin (MSA), as well as the formation of a stable complex with the antigen.

Nonpurified adiponectin, which can only be expressed in eukaryotic cells and typically at low yields, was used to assess the versatility of the developed platform. The protein was tagged with an N-terminal FLAG epitope and expressed as a secreted protein, and the resulting medium was used as the selection antigen. Then, nanobody-expressing yeasts were identified with a fluorescently labeled anti-FLAG antibody. Binding to adiponectin of three unique clones, obtained after four selection rounds, was confirmed by SPR.

Finally, the authors carried out a selection of Nbs against the active conformers of β2AR and A2AR. For β2AR, several Nbs specific for the antagonist-binding receptor conformation were obtained, with affinities between 44–151 nM. For A2AR, two conformer-specific Nbs were also obtained.

### 4.3. Zimmermann et al., (2018)

To design their synthetic library, Zimmermann and coworkers [14] carried out a structure-based analysis of a set of Nb crystal structures, focusing on the shape of the surface created by the binding region, which greatly depends on the length of CDR3. They noted that Nbs with a short CDR3 (six aa.) show a concave shape, those with an intermediate length (12 aa.) show a protrusion which they called Loop, while those with a longer length (16 aa.) present a convex surface. From this study, they selected several previously reported Nbs as templates to construct a library for each of the binding site shapes.

*Framework selection*. For both the concave and loop libraries, a common consensus framework derived from the GFP-binder 3K1K nanobody [64] and the β2-adrenergic receptor binding Nb 3P0G [65] was used as a template. For the convex library, the lysozyme binding Nb 1ZVH [66] was selected, based on its extended hydrophobic core that favors a bent conformation of CDR3. To check the stability of the two frameworks, serine and threonine mutants were generated in positions selected for randomization, leaving the hydrophobic core invariant, as well as amino acids, such as proline that impose conformational restraints in the CDRs. As result, the mutant Nbs showed a considerable increase in Tm (by 21–35 °C) compared to their precursors.

*CDR design*. The CDR randomization strategy focused on obtaining an optimal balance between charged, polar, aromatic, and non-polar amino acids, aiming to achieve an overall moderate hydrophobicity on the randomized surface. It has been shown that lowering the hydrophobicity of the variable region counteracts the enrichment of sticky binders when selecting against membrane proteins [67] and decreases the propensity for aggregation, a strategy also adopted by the authors of the NaLi-H1 library [12]. For CDR synthesis, three different mixtures of trinucleotides were used for the randomized positions, depending on their location: (i) in loops, (ii) in the transitions from loops to β sheets, and (iii) in the middle of β sheets. The amino acid composition of each mixture was carefully tailored based on the observed natural amino acid frequency at the three different locations. The estimated theoretical diversity was 8.3 × 10^17^, 4.3 × 10^19^ and 2.8 × 10^22^ for the concave, loop, and convex libraries, respectively.

*Library construction*. The DNA fragments containing the CDRs were generated by assembly PCR and ligated in two subsequent steps using Type IIS restriction enzymes. The three libraries were expressed using ribosome display. This technique involves more complex procedures as compared to phage or yeast display, but instead allows a larger library size (9 × 10^12^ in this study [14]), and therefore, a higher clonal diversity. It is worth noting that the authors of this study recently published a detailed protocol that combines the enormous experimental variety of the ribosome display with the versatility and ease of use of phage display [14,68].

*Library validation*. The library was tested against four different antigens. Soluble maltose-binding protein (MBP), considered an easy molecular target, was the first choice (Table 2). Several Nbs were obtained from each library, with affinity values reaching 0.5 nM. The crystallographic structures of three Nbs derived from the convex library showed a high structural similarity with the precursor Nb (1ZVH), corroborating the importance of the hydrophobic core in shaping the variable region. Furthermore, the interaction patterns were similar in the Nb-MBP complexes, both in position and amino acid type.

In a second test against the membrane protein ABC transporter TM287/288, the standard selection protocol was not sufficient to obtain binders (Table 2). The authors identified several bottlenecks: (i) PCR amplification of cDNA to retrieve the output from the initial round of ribosome display and (ii) phage infection of *E. coli* to retrieve the output from the first phage display selection round. Modification of the selection protocol [14,68] resulted in a high number of positive ELISA hits and high affinities (up to ~2 nM). Nbs were selected also against the outward-facing (OF) TM287/288 forms, using as target a mutant (TM287/288(E517A)) that prevents ATP hydrolysis. A high number of binders selective for the ATP-bound OF TM287/288(E517A) mutant were recovered, and one of these Nbs showed an IC50 of 62 nM against the native TM287/288 transporter.

Finally, Nbs were selected against two of the human SLC transporters: equilibrative nucleoside transporter 1 (ENT1) and glycine transporter 1 (GlyT1), both involved in various diseases [67] (Table 2). According to the authors, multiple previous attempts to generate mouse antibodies or nanobodies against these targets by immunization had failed, presumably due to the low thermal stability of these proteins and the limited number of accessible epitopes. Strikingly, at least one Nb from each library stabilized the inhibited conformation of GlyT1 with affinities ranging from 3 µM to 0.5 nM and increased the thermal stability of the complex. For ENT1, only one Nb was obtained, in this case from the concave library, with a 40 nM affinity. These results indicate, as with the NaLi-H1 library [12], that rational design of the CDRs and inclusion of various CDR3 lengths is a solid strategy to obtain high affinity synthetic Nbs against a wide variety of antigens.

### 4.4. Chen et al., (2021)

Chen and coworkers [15] designed a platform called CeVICA (Cell-free VHH Identification using Clustering Analysis) that integrates a library of synthetic Nbs, ribosome display and computational prediction of binders using CDR-directed clustering analysis. By difference with the studies discussed so far, this work not only includes the design of the synthetic library, but also a protocol comprising a computational component and a phase of in vitro affinity maturation. As for the previous examples, the design was based on sequence and structural analysis of natural Nbs, in this case of a set of 1030 sequences and 298 crystallographic structures.

*Framework design*. A combinatorial scaffold was designed, composed of several versions of the different framework segments. So, the combinatorial scaffold was composed of three versions of frame1, one of frame2, three versions of frame3 and one of frame4. The three versions of frame1 and frame3 were derived from (i) the consensus sequence extracted from the analyzed set of natural Nbs; (ii) the natural Nbs A310 [26]; and (iii) a GFP-binding nanobody [64], respectively.

*CDR design*. Compared to previous studies [12,13,14], the CDR design for this library is slightly different regarding the chosen CDR lengths and the randomization strategy. One notable difference is the use of all the amino acids, including cysteine, at all randomized positions. The chosen lengths were: 7 for CDR1, 5 for CDR2 and 6, 9, 10, or 13 for CDR3, which according to their study match the most frequent CDR lengths observed in natural Nbs. Finally, a diversity of 3.7 × 10^11^ complete sequences was obtained using ribosome display.

*Library validation*. Nbs were selected against two antigens: EGFP and the receptor-binding domain (RBD) of the spike protein of SARS-CoV-2 (Table 2). After three selection rounds, the Nbs obtained against both antigens were clustered based on their CDR similarity. Clusters containing Nbs for the two antigens were excluded from further analysis, assuming a non-specificity, and then the study continued only with the RBD binders. This clustering analysis, although at the cost of sacrificing possible binders, allows reducing the sampling space, making the process faster and less resource demanding. As result, 14 Nb clones were obtained, of which three were strong, eight weak and three non-binders. Six of these Nbs (three strong and three weak binders) showed >30% inhibition of the infection at 1 μM in neutralization assays using a SARS-CoV-2 Spike-pseudotyped lentivirus. Finally, after an in vitro maturation process based on error-prone PCR, the most potent neutralizer showed an IC50 of 62.7 nM, comparable to antibodies identified in human patients [69,70].

### 4.5. Other Synthetic Libraries

We have focused our review on a handful of synthetic libraries, which we selected because of their particular design and validation strategies. Other synthetic libraries have been designed following similar or simpler strategies. Thus, Yan and coworkers built a library in which only CDR3 was randomized [16], while Sevy et al., followed a strategy similar to that described by Chen and coworkers [15], basing their design on the sequence and structural computational analyses [17]. Ferrari et al. focused their work on the design of a scaffold optimized for bacterial expression and suitable for the construction of ribosome display libraries [21]. Warner et al., on the other hand, centered their work on the generation of synthetic Nb libraries derived from grafting experiments for subsequent in vitro affinity maturation [19]. As the last, very recent example, Zhao and coworkers developed a unique and interesting alternative for the expression of synthetic libraries in mammalian cells [18]. In their study, two methods were proposed to incorporate randomized CDRs by PCR and cellular transfection into double-stranded DNA (dsDNA). This technology allows obtaining ~10^6^ unique sequences in mammalian cells, without the need for plasmid transformation-extraction and lentivirus preparation-infection, which considerably reduces the variability of plasmid-based libraries.

### 4.6. Impact of Synthetic Nb Libraries

During the last two decades, synthetic VHH or Nb libraries have been finding their way in a field dominated by immune libraries, with notable advances made during the last 5 years, mainly in terms of the quality of the selected nanobodies. Their impact is also reflected by the important number of citations accumulated so far (December 2021) for the four libraries analyzed here [12,13,14,15].

It is difficult to track in the literature how many nanobodies selected from these libraries have been reported, and their applications. In this regard, it is worth mentioning the commercial application of the NaLi-H1 library, protected by a patent (WO/2015/063331) that covers the scaffold and the implemented CDR variability. A nanobody selection and validation service based on this library is offered by Hybribody (Hybrigenics Services, Evry, France) https://www.hybribody.com/contenu/synthetic-vhh-library-menu/our-synthetic-antibody-library (accessed on 26 December 2021). Its commercial orientation might result in lower exposure of the derived Nbs in the literature [71]. On the other hand, several Nbs derived from the libraries described by Zimmermann and coworkers [14] have been reported. These studies essentially include Nbs directed against membrane proteins, including the RDB domain of SARS-CoV2 [72,73,74]. Similarly, the library designed by McMahon and coworkers has been widely used, including also the selection of Nbs against the spike protein of SARS-CoV2 [75], chaperone Nbs to solve proteins by X-ray crystallography and cryo-EM [76], among others [77,78,79].

## 5. General Discussion

So far, the design of synthetic Nb libraries has been strongly influenced by the wide representation of llama Nbs. Although works based on camel and dromedary Nbs are also reported, these are no longer as common. Most likely because llamas are cheaper to maintain than camels or dromedaries. In addition, studies of semisynthetic libraries based on single domain antibodies from sharks have been reported [9,10,11]. Overall, the ease of obtention, the number of reported sequences, as well as the higher Tm of llama-derived Nbs, make them more attractive for Nb engineering [48].

The development of a stable universal framework dates from 2005 [24] and since then this framework has been used in several studies as a scaffold for synthetic libraries [16,19,23]. In recent years, novel strategies have been developed to obtain highly stable frameworks which not only can support a variety of CDR lengths but can also function in reducing environments, like the cytoplasm [12]. In addition, these frameworks have been humanized without loss of solubility, stability, or affinity [12,15]. A common strategy has been to generate a consensus framework from sequences of natural Nb repertoires [12,13,15,17,21]. In other cases, previously reported stable Nbs have been used as templates [14,18,19]. In addition, several engineering strategies have been devised to improve the physicochemical properties of the Nbs [8,26,27,29,30,39].

CDR design, on the other hand, has followed strategies having common elements, but differing in practice from one study to another. CDR1 and CDR2 have been randomized intending to recapitulate the amino acid diversity of natural repertoires [13,15] or have simply been randomized partially or completely [14]. In addition, a strategy that favors intrinsic solubility has been implemented [12]. The design of CDR3 has followed different criteria. Thus, Moutel et al. completely randomized each position with all the amino acids except cysteine [12], Chen et al. followed the same approach but included cysteines [15], while McMahon and coworkers excluded both cysteine and methionine [13]. On the other hand, Zimmermann et al. tuned their CDR3 randomization focusing on keeping the geometric shape of the Nb binding site, which depends on CDR3 length [14].

Amino acid variability is commonly implemented using degenerate codons [80]. NNS or NNK degeneracy can be used to encode the 20 natural aa, obtaining a total of 32 codons. Although this method is economically more affordable, it has a few disadvantages. One of them is the appearance of stop codons, which produce truncated, non-functional molecules. Furthermore, the high codon redundancy is biased toward certain aa. On the other hand, it is possible to tailor randomization by playing with different degenerate codons that yield different sets of aa, but even this way it is difficult to avoid the occurrence of unwanted amino acids.

These drawbacks can be overcome using a more precise, although more expensive method: trinucleotide DNA assembly, as employed by Moutel and coworkers [12]. Its main advantage over the use of degenerate codons is that specific amino acids can be encoded, allowing streamlined randomization with precise control of the desired amino acids at each position.

A very important issue in the construction of a synthetic Nb library is the expression system. Currently, phage display is the most common, but other systems are being also employed, such as ribosome display and cell display, including yeast and bacterial display, and more recently, the expression on mammalian cells [18]. These systems vary in complexity, allowing different library sizes and experimental possibilities.

Considering the non-specificity of synthetic libraries towards any particular antigen and, therefore, the requirement of a large library to guarantee the largest possible variability, then ribosome display is probably the recommended option since it allows library sizes in the order of 10^12^ clones. However, this expression system requires experience to implement elaborated protocols [69]. Phage display allows library sizes in the order of 10^9^, that is, three orders of magnitude smaller than what can be obtained with ribosome display. On the other hand, phage display has several advantages that have made this system the most popular among researchers. This technique has been widely used for expressing libraries of recombinant peptides and many different proteins, due to its ease of implementation. Recently, Zimmermann and coworkers proposed a protocol that combines the first round of selection from ribosome display to take advantage of the high variability, followed by selection rounds based on phage display, taking advantage of its ease of use [14,68]. Yeast display produces an approximate variability of 10^8^, however, its main advantage is that it allows classifying the selected clones by flow cytometry [81], which has made this technique also popular. Each expression system has advantages and disadvantages, so researchers must first assess which of them best suits their needs.

### 5.1. Theoretical vs. Experimental Variability

The design of a synthetic Nb library represents a protein engineering challenge, not only because of the required physicochemical properties of the resulting Nbs but also because of the enormous variability that a library intends to mimic in an efficient way. If we consider 20 CDR positions to randomize, using all amino acids, the theoretical number of combinations would be 20^20^≈10^26^. Considering that the maximum number of unique sequences that can be obtained experimentally is in the order of 10^12^, then the theoretical and the achievable diversity would differ by 14 orders of magnitude. Tailored randomization may reduce this difference by several orders, but still, the two repertoires would greatly differ.

The huge difference between the theoretical and experimental sizes implies, for example, that if a library is constructed many times using the same CDR design, totally different sub-libraries would be obtained every time. Furthermore, and following this reasoning, randomization using sets of naturally occurring amino acids, even if these sets are tailored for each specific CDR position, does not guarantee that the resulting library will mimic a natural repertoire since this is defined not by the presence of certain amino acids at each individual CDR position, but by the specific amino acid combinations that in whole shape the binding site.

Nonetheless, what is most important is to obtain a library with rich diversity, able to provide good binders against a large variety of antigens. As highlighted in this review, a proper framework selection together with an elaborated CDR design can lead to the selection of stable, high affinity nanobodies against different antigens, which, if necessary, might be further improved by in vitro affinity maturation.

### 5.2. Concluding Remarks

Nanobodies are becoming more and more popular as versatile molecules that have found their way in a large variety of applications. Currently, immune libraries are overwhelmingly the main source of nanobodies, and according to the Clinical Trials website (https://clinicaltrials.gov/ (accessed on 19 December 2021)), so far none of the Nbs in different phases of clinical studies have been derived from synthetic libraries. However, this scenario might change in the future, as synthetic libraries continue to prove their value as alternative sources of stable, high affinity Nbs, with lower costs and higher speed. Remarkably, one synthetic library (NaLi-H1) is already being exploited in a commercial setting, paving the road for similar entrepreneurships. In the coming years, we foresee the advent of many more synthetic Nb libraries, both in academic and commercial settings, which will become a main source of nanobodies for multiple applications.

## Figures and Tables

**Table 1:** Synthetic and Semi-synthetic libraries.

Name ^a^	Framework Source	CDR Lengths	CDR Randomization Strategy	Library Size/Display System	Reference
NaLi-H1	Llama-derived (consensus sequence from immune Nb set)	CDR1: 7 CDR2: 7 CDR3: 9, 12, 15, 18	CDR1 and 2: fully randomized. Partially recapitulates the natural diversity, with the inclusion of polar amino acids. CDR3: fully randomized	3 × 10^9^ Phage Display	[12]
McMahon et al.	Llama-derived (consensus sequence derived from llama genes)	CDR1: 7 CDR2: 5 CDR3: 7, 11, 15	CDR1 and 2: 4 and 1 highly variable positions, respectively CDR3: fully randomized, including variable adjacent positions	1 × 10^8^ Yeast Display	[13]
Concave	Dromedary-llama (consensus framework between dromedary-derived 3K1K and llama-derived 3P0G.)	CDR1: 7 CDR2: 6 CDR3: 6	CDR1 and 2: 5 positions were rationally randomized CDR3: 5 positions were rationally randomized	9 × 10^12^ Ribosome Display	[14]
Loop	Dromedary-llama (consensus framework between dromedary-derived 3K1K and llama-derived 3P0G.)	CDR1: 7 CDR2: 6 CDR3: 12	CDR1 and 2: 5 positions were rationally randomized CDR3: 6 positions were rationally randomized	9 × 10^12^ Ribosome Display	[14]
Convex	Dromedary-derived (from Nb 1ZVH)	CDR1: 7 CDR2: 6 CDR3: 16	CDR1 and 2: 4 positions were rationally randomized CDR3: 10 positions were rationally randomized	9 × 10^12^ Ribosome Display	[14]
CeVICA	Consensus framework and reported natural Nbs: A310 and GFP-binding nanobody	CDR1: 7 CDR2: 5 CDR3: 6, 9, 10, 13	CDRs: All positions were fully randomized, including cysteine	3.68 × 10^11^ Ribosome Display	[15]
Yan et al.	cAbBCII10	CDR3: 16	CDR3: fully randomized	1.65 × 10^9^ Phage Display	[16]
Alp_LowDiv, Hum_LowDiv, Alp_HighDiv, Hum_HighDiv	Alpaca-derived and partially humanized (consensus sequence derived from alpaca genes)	CDR1: 8 * CDR2: 7 * CDR3: 6–18	CDR1 and 2: All (for Alp_HighDiv, Hum_HighDiv) or selected (for Alp_LowDiv, Hum_LowDiv) positions were randomized CDR3: fully randomized	1.2 × 10^9^, 1.5 × 10^9^, 0.9 × 10^9^, 1.1 × 10^9^ Yeast Display	[17]
Zhao et al.	GFP-binding nanobody (cAbGFP4)	CDR1: 9 * CDR2: 8 * CDR3: 7	All CDRs were fully randomized	~1 × 10^6^ Mammalian Cells	[18]
Wagner et al.	dromedary-derived (3K1K)	CDR1: 9 CDR2: 6 CDR3: 18	CDR2 and 3: Randomization of selected positions recapitulating natural diversity and enriching with amino acids of the same nature	9 × 10^9^ Phage Display	[19]
Chi et al.	cAbBCII10-derived	CDR1: 8 CDR2: 8 CDR3: 18	All CDRs were fully randomized	1.2 × 10^10^ Phage Display	[20]
Ferrari et al.	Llama-derived (consensus framework from annotated sequences)	CDR1: 9 * CDR2: 7 * CDR3: 16	CDRs: All positions were randomized recapitulating the natural diversity and excluding cysteine	~1 × 10^12^ Ribosome Display	[21]
Stefan et al.	NaLi-H1 framework derived	CDR3: 9, 12, 15	All CDRs were fully randomized	3.18 × 10^10^ Phage Display	[22]
Wei et al.	cAbBCII10	CDR1: 8 CDR2: 8 CDR3: 9–20	CDR1 and 2: Partially randomized CDR3: fully randomized	1 × 10^12^ Phage Display	[23]
Liu et al.	SPSL1 naïve library	CDR3: 13, 16, 18	CDR3: fully randomized	1 × 10^9^ Phage Display	[10]
Könning et al.	naïve bamboo shark scaffolds	CDR3: 12, 14, 16, 18	CDR3: fully randomized	3 × 10^9^ Yeast Display	[9]

^a^ Library name or authors; * Not explicitly defined in the article.

**Table 2:** Antigens employed to validate the functionality of synthetic libraries.

Library	Antigen	Antigen Features	Pannings	K_D_ (M)	Ref.
NaLi-H1	EGFP mCherry tubulin β-actin p53 (83 first aa fragment) HP1α RHO GTPase HER2	Soluble protein Soluble protein Polymeric protein Polymeric protein Soluble protein Soluble protein Membrane protein/specific conformer Membrane protein/surface antigen	2–4	1.6 × 10^−10^–4 × 10^−9^	[12]
McMahon et al.	HSA Human Adiponectine β_2A_R A_2A_R	Soluble protein Soluble protein Membrane protein/specific conformer Membrane protein/specific conformer	4	44 × 10^−9^–430 × 10^−9^	[13]
Concave, loop, and convex	MBP ABC transporter TM287/288 ENT1 GlyT1	Soluble protein Membrane protein/specific mutant Membrane protein/specific conformer Membrane protein/specific conformer	3	4.94 × 10^−10^–2.5 × 10^−6^	[14]
CeVICA	EGFP RBD	Soluble protein Soluble protein	3	2.18 × 10^−9 a^	[15]

^a^ After in vitro affinity maturation.

## Data Availability

Not applicable.
